# Effects of mind-body exercises on cognitive impairment in people with Parkinson's disease: A mini-review

**DOI:** 10.3389/fneur.2022.931460

**Published:** 2022-09-01

**Authors:** Ting Zhang, Wei Liu, Song Gao

**Affiliations:** ^1^College of Physical Education and Health Sciences, Zhejiang Normal University, Jinhua, China; ^2^University Hospital, Zhejiang Normal University, Jinhua, China; ^3^Physical Education College, Guangxi University of Science and Technology, Liuzhou, China

**Keywords:** Parkinson's disease, cognitive impairment, mind-body exercise, rehabilitation, limitations

## Abstract

Parkinson's disease (PD) is an important health problem caused by the degeneration of brain neurons. Bradykinesia and lower balance ability seriously affect the quality of life of people with PD. Non-motor symptoms, such as cognitive impairment, accompany the course of the disease but still lack sufficient attention. In general, drugs combined with cognitive training are the most common ways to improve cognitive impairment in people with PD. However, long-term use of psychiatric drugs may lead to side effects such as brain death and movement disorders. Recently, mindfulness has been used by researchers in the treatment of cognitive impairment, because healthy older adults who engage in mind-body exercises for a long time have higher cognitive levels than normal aging populations. Mind-body exercise, as a therapy that combines concentration, breath control, and physical activity, is beneficial for improving practitioners' brain and mental health. Mind-body exercises such as Tai Chi, yoga, dance, and Pilates can improve cognitive performance in older adults with or without cognitive impairment. Therefore, mind-body exercise may be a feasible strategy for the treatment of cognitive impairment in people with PD. This study summarizes the latest evidence that mind-body exercises including Tai Chi, Qigong, yoga, and dance improve cognitive impairment associated with PD. We also explored the limitations of current mind-body exercise research, aiming to provide new ideas for improving mind-body exercise as a strategy to alleviate cognitive impairment in people with PD.

## Introduction

Parkinson's disease (PD) is a prevalent neurodegenerative disease in the United States, second only to Alzheimer's disease in the incidence (1). Worldwide, approximately nine million people over the age of 50 are expected to have PD by 2030 (2). As the disease worsens, people with PD develop different degrees of motor dysfunction, which seriously affects activities of daily living and quality of life. Most interventional studies have focused on improving motor ability, as it is a typical symptom of PD. However, non-motor symptoms such as psychiatric symptoms, autonomic dysfunction, and cognitive dysfunction also accompany the entire process of PD but are often overlooked (3). Eighty percentage of people with PD develop cognitive impairment over time (4).

Mild cognitive impairment is common in persons with PD, which often progresses to Dementia with Parkinson's disease (5). Cognitive impairment can occur at any stage of PD and often precedes the diagnosis of PD (2). Cognitive impairment associated with PD is mainly manifested as memory impairment, disorientation, numeracy impairment, and language impairment (6). It has been reported that the incidence of cognitive decline in PD patients with a history of more than 15 years reaches 85%, which seriously affects their quality of life and social function (7). Cognitive impairment is irreversible, and effective interventions for dementia are limited. Therefore, preventing mild cognitive impairment and improving cognitive function is essential to delaying or preventing Dementia with Parkinson's disease.

Currently, although there is no cure for dementia or to alter its progression, early targeted interventions can delay cognitive decline (6). There is a lack of high-quality evidence that pharmacotherapy can improve cognitive function or delay cognitive decline in patients with mild cognitive impairment (8). Medications that improve cognition generally belong to the category of psychiatric drugs, and long-term use may lead to side effects such as brain death, cognitive impairment, and motor impairment (9). Therefore, non-drug therapies such as exercise, music, and acupuncture have received increasing attention (10). In addition, psychoactivity and physical activity are encouraged as non-pharmacological interventions to reduce cognitive risk to improve cognitive performance in older adults with and without cognitive impairment.

Mind-body exercise, as a therapy that combines mental focus, breath control, and physical activity, is beneficial for improving brain and mental health (11). Previous meta-analyses have shown that mind-body exercises such as tai chi, yoga, dance, and Pilates can improve cognitive performance in older adults with or without cognitive impairment (12). Mind-body exercises such as yoga, tai chi, and qigong are growing in popularity in the United States. Clarke et al. (13) found that more than 10% of adults in the United States have tried mind-body exercises. Previous studies have shown that mind-body exercises can effectively improve PD's motor symptoms, while less attention has been paid to non-motor symptoms, especially those associated with cognitive impairment.

This study summarizes the latest evidence that mind-body exercises improve PD with cognitive impairment, including Tai Chi, Qigong, yoga, and dance. We also explored the limitations of current related research, aiming to provide new ideas for improving mind-body exercise as a strategy to alleviate cognitive impairment in people with PD. This review suggests that mind-body exercise may be a valuable and feasible approach to enhancing cognitive impairment in PD.

## Mind-body exercises

Mind-body exercises control physical and mental activities with mind or self-awareness and focus attention to maintain control (14). Practitioners put a lot of effort into making mind-body movements to make sure that the mind does not wander or leave in the process. It is important to maintain proper breathing and posture during mind-body exercises, through which body control and awareness are trained. Relaxed breathing and muscle relaxation can also be used as a mind-body exercise, where the practitioner focuses his or her mind on patterns of breathing and muscle tension to control the way he or she responds to various stimuli (15). In attention-based mind-body interventions (including mindfulness, meditation, guided imagery, and hypnosis), a high level of attention is required to draw the mind to certain thoughts or imagery with soothing movements. Movement-based mind-body movement, through a series of controlled movement and concentration exercises, enhances mind-body coordination and awareness. This article collects evidence that common mind-body exercises improve cognitive impairment in PD. Characteristics of the included studies are shown in Table 1.

**Table 1:** Characteristics of the included studies.

**References**	**Participants**	**Interventions**	**Outcomes**
Guan and Li (18)	*n* = 60 Age: 55–82 y HY scale 2–3 stage ability to walk with/without aids	Yang style Tai Chi vs. routine medication 60 min, 4 times/week, 12 weeks	MoCA^*^, FAQ^*^
Wu et al. (20)	*n* = 52 Age: 45–75 y HY scale 1–3 stage ability to walk with/without aids	Yang style Tai Chi vs. routine medication and exercise recommendations 40 min, 4 times/week, 16 weeks	MoCA^*^, PDQ-39^*^
Nocera et al. (24)	*n* = 21 Age: 60–80 y HY scale 1–3 stage ability to walk with/without aids	Yang style Tai Chi short form vs. no intervention 60 min, 3 times/week, 16 weeks	Cognitive-executive function, PDQ-39^*^, TFES
Moon et al. (34)	*n* = 17 Age: 40–80 y HY scale 1–3 stage ability to walk with/without aids	Qigong vs. physical activities 20 min, 2 times/day, 12 weeks	PDSS-2, GAI, GDS, 16-item Parkinson Fatigue Scale, Cognitive functions, NMSQ, PDQ-39
Cheung et al. (48)	*n* = 20 Age: 49–75 y HY scale 1–3 stage ability to walk with/without aids	Hatha yoga vs. no intervention 60 min, 2 times/week, 12 weeks	UPDRS-III, MoCA, BDI
Hashimoto et al. (55)	*n* = 46 Age: 50–80 y HY scale 2–4 stage ability to walk with/without aids	Dance vs. PD exercise vs. no intervention 60 min, 1 time/week, 12 weeks	Motor function^*^, Cognitive function, Mental symptom, General PD symptoms
Kalyani et al. (56)	*n* = 49 Age: 40–85 y HY scale 1–3 stage ability to walk with/without aids	Dance vs. no intervention 60 min, 2 times/week, 12 weeks	Cognition, UPDRS-I, UPDRS-II, PDQ-39
Michels et al. (57)	*n* = 13 Age: 50–70 y HY scale 1–3 stage ability to walk with/without aids	Dance vs. no intervention 60 min, 1 time/week, 10 weeks	UPDRS, MoCA, BDI, BBS, TUG, PDQ-39^*^
Rios Romenets et al. (58)	*n* = 40 Age: 49–80 y HY scale 1–3 stage ability to walk with/without aids	Tango vs. no intervention 60 min, 2 times/week, 12 weeks	MoCA, BDI

* indicates statistically significant differences between the intervention group and the control group (*p* < 0.05).

HY scale, Hoehn and Yahr scale; MoCA, Montreal Cognitive Assessment; FAQ, Functional Activities Questionnaire; PDQ-39; TFES, Tinetti's falls efficacy scale; PDSS-2. Parkinson's disease sleep scale-2; GAI, Geriatric anxiety index; GDS, Geriatric depression scale; NMSQ, Non-motor symptom questionnaire; BDI, Beck depression inventory; UPDRS, the Unified Parkinson's Disease Rating Scale; BBS, Berg Balance Scale; TUG, timed up and go.

## Tai Chi

Tai Chi originated in China and had a wide range of mass foundations, especially among elderly groups. Tai Chi is a combination of physical and mental. Its movement mode is slow and smooth. The rhythmic breathing mode and body control are conducive to alleviating the practitioner's pressure and regulating bad emotions (16). In simple terms, Tai Chi is a composite movement of aerobic exercise and psychological hints. However, Tai Chi has its special diagonal mode. Studies have shown that Tai Chi can effectively improve cognitive functions such as the elderly's attention, memory, execution, and response-ability, thereby improving their ability to participate in social activities (17). The current research is mostly concentrated on Tai Chi training or other aerobic exercises on PD's motor and balance ability, ignoring cognitive impairment with PD.

A research study by Guan and Li (18) found that Tai Chi training can effectively improve PD patients' cognitive impairment. They randomly divided 60 mild cognitive impairment persons with PD into a Tai Chi group and a control group. The tai chi group adopted simplified 24-style tai chi, and the control group only received conventional drug treatment. After a 12-week Tai Chi intervention, PD patients' Montreal Cognitive Assessment (MoCA) scores, memory function, executive function, attention, and computing power were significantly improved, while the control group showed no significant difference. Tai Chi is considered a moderate-intensity aerobic exercise. Aerobic exercise can change the structure and function of the prefrontal, parietal, and temporal lobes of the brain, and the frontal lobe has functions such as memory renewal, attention switching, and multitasking coordination (19). This is similar to the findings of Wu et al. (20), who found the effect of 24-style Tai Chi on cognitive function and health-related quality of life in mild cognitive impairment patients with PD. The results showed that the Tai Chi group had significantly higher MoCA scores and significantly lower PDQ-39 scores, while the control group who received anti-Parkinsonian medication alone did not delay the deterioration of cognitive impairment. Its mechanism may be related to Tai Chi's special movement pattern. The diagonal movement pattern of Tai Chi movements is particularly prominent, which is similar to the proprioceptive neural facilitation technique (a training method that enhances neuromuscular responses with diagonal and spiral movements of the limbs and trunk), For example, the movement of the White Crane Spreads its Wings is very similar to the D1 flexion (flexion/adduction/external rotation) pattern of the upper limbs; Grasp the Bird's Tail closely resembles the inverse motor pattern of the upper and lower limbs (21). The upper and lower limbs' diagonal spiral movement pattern can effectively improve the arousal degree of the central nervous system, thereby improving the motor control ability and cognitive level (22). Another study by Lam et al. (23) showed that 1-year tai chi training not only significantly improved balance ability in older adults but also improved older adults' concentration at risk of cognitive decline. They hypothesized that in addition to being a form of physical activity, tai chi involves memory training of motor sequences, coordination between attention, and voluntary motor control. However, a study by Nocera et al. (24) showed that 16-week of Tai Chi training did not improve cognitive-executive function in persons with PD. The findings suggested that Tai Chi may not be an ideal model for improving cognitive-executive function in PD patients. However, this may be related to the small sample size included in their study. For example, compared to the control group, the Tai Chi group did improve on measures such as attention and working memory, in addition, these proliferations did not reach statistical significance.

Long-term tai chi practice can thicken the right precentral gyrus, insular sulcus, middle frontal sulcus, and left superior temporal gyrus, which means that regular tai chi practice significantly increases the whole brain volume and delays brain function decline (25). Raising heart rate can increase blood flow to the brain, glucose, and oxygen supply, and provide an energy basis for the rapid mobilization of neurons in the brain. During Tai Chi practice, the average heart rate of practitioners can be increased to 121 beats /min, which is the optimal heart rate range for improving cognitive function (26). Besides, the combination of Tai Chi movements and thoughts can strengthen the brain's ability to regulate and control the organs of the whole body (27). To sum up, Tai Chi is popular among the elderly because of its slow rhythm and moderate intensity. As one of the physical and mental exercise therapy, Tai Chi can be used as an auxiliary medical method to improve the cognitive function and quality of life of people with PD.

## Qigong

Qigong originated in China and had a history of thousands of years. Qigong is a mind-body exercise rooted in traditional Chinese medicine and consists of a harmonious series of meditation, deep breathing, and gentle body movements (28). To standardize the form and promotion of Health Qigong, the General Administration of Sports in China has released 11 categories of Health Qigong, such as Yijinjing and Baduanjin (29). Studies have shown that Qigong can effectively treat or delay various diseases, such as depression, insomnia, poor balance, and cognitive dysfunction (30). Therefore, the effect of Qigong on non-motor symptoms, especially cognitive impairment, in people with PD deserves further investigation.

The study by Tsang et al. (31) included frail elderly people in Hong Kong who were randomly divided into a Qigong group and a control group. The Qigong group underwent a 12-week Qigong program, while the control group participated in the newspaper reading program at the same time and frequency. The research results showed that Qigong exercise can effectively improve frail elderly people's thinking and decision-making ability. Participants in the Qigong group showed significant improvements in their thinking and decision-making abilities, while other cognitive function aspects, such as memory and attention, did not show any significant changes after practice. The findings initially supported the positive benefits of practicing Health Qigong on specific aspects of cognitive function in frail older adults. Notably, gender bias was a major limitation of the study, which was dominated by women. In addition, the researchers did not further explore the relationship between practicing Qigong and maintaining its effects. Another study by Cai and Zhang (32) reported that after a 24-week intervention, the time of Wechsler's logical memory test and digital memory span test in the Qigong group decreased significantly compared with the control group, and the total time score decreased with the prolongation of the intervention time, suggesting that Health Qigong can improve memory capacity in elderly patients with mild cognitive impairment. Health Qigong practitioners need to maintain a higher degree of concentration when learning relevant movements in the early stage, to better memorize and imitate the coach's movements. Thus, the subjects started to imitate the action from vision, and gradually form an image in the brain through accumulation, thereby improving their memory ability. A systematic review and meta-analysis of 16 randomized controlled studies indicated that Baduanjin combined with conventional therapy significantly improved cognitive and memory function in patients with mild cognitive impairment, and its efficacy was superior to conventional therapy (33). The study by Moon et al. (34) assessed cognitive function in people with PD after Qigong intervention with a positive assessment battery, 10 o'clock drawing test, and follow-up making tests A and B. However, the results found that Qigong only relieved insomnia symptoms in people with PD, and did not find a significant effect on the outcome measures related to cognitive function.

To sum up, Health Qigong can effectively improve the cognitive ability of patients with mild cognitive impairment and delay the deterioration of the disease. Health Qigong is less restricted by educational level, equipment, and venue. It can be used as an auxiliary rehabilitation method for the elderly with cognitive impairment and has promotion value. However, there seems to be no conclusive evidence for the contribution of Qigong to improving cognitive impairment in people with PD. This may be related to the small sample size of the included subjects. Furthermore, the currently promoted Health Qigong is only suitable for elderly individuals with healthy or mild cognitive impairment and is not suitable for PD patients with cognitive impairment. In the future, more research on Health Qigong intervention in PD with mild cognitive impairment should be added, and more targeted improved Health Qigong forms should be explored.

## Yoga

Yoga, which originated in ancient India in the 3rd century BC, is classified by the National Institutes of Health as a form of complementary and integrative medicine, which includes the practice of specific postures (asanas), regulating breathing (pranayama), and meditation (35). Yoga practice integrates the physical, mental, and spiritual components of an individual to improve health and wellbeing (36). Yoga is considered a mind-body therapy and is widely used among American adults with neurological disorders (37). Mind-body interventions including yoga, tai chi, mindfulness meditation, and qigong have shown promising results in improving cognitive impairment associated with aging (38, 39). A recent study by Gothe et al. (40) compared the effects of 8-week hatha yoga and intensive stretching on cognitive function in 118 community-based older adults. The findings showed that the yoga group significantly improved executive function, working memory, mental transfer efficiency, and flexibility. A meta-analysis and systematic review showed that yoga improves cognitive and working memory performance in healthy individuals (41). Yoga has positive effects on some neurological diseases, such as stroke sequelae, Parkinson's disease, Alzheimer's disease, etc. (42).

A 6-week study by Bonura and Tenenbaum showed that yoga improved anxiety, general self-efficacy, and self-efficacy in daily life in older adults, thereby promoting the mental health (35). Although the study was brief, the results suggested that yoga has the potential to improve mental health in older adults. While yoga contains an element of physical activity, it has a greater impact on mental health than just an alternative physical activity form. Physical activity is an integral part of yoga, but it is only a means to better understand the mental processes of the participants. Depression, a potential risk factor for dementia, can be treated with yoga as an effective and cost-effective complementary treatment (43). A study by Eyre et al. (44) showed that Kundalini Yoga significantly improved depression in patients with mild cognitive impairment. Notably, in addition to weekly yoga sessions of up to 60 min, the researchers also asked subjects to meditate for 12 min a day as their homework. However, the practice effect of yoga may have influenced the observed improvements. Furthermore, these findings suggested that improved memory is associated with increased default mode network connectivity in the anterior, posterior and medial prefrontal regions (44). Yoga is thought to exert its effects by reducing stress, reducing inflammation, enhancing neuroplastic processes (e.g., brain-derived neurotrophic factor production), increasing antioxidant levels, and increasing telomerase activity (45, 46). Another randomized controlled trial yielded similar results (47). However, the study by Cheung et al. (48) found that 12-week yoga training can improve PD patients' motor function, physical activity, and quality of life, but did not show non-motor symptoms such as cognitive impairment in people with PD significant advantage. This may require further studies with larger sample sizes to determine its effect on non-motor symptoms.

In various studies, subjects were generally asked to go home for a meditation practice after intensive yoga training. Clinicians advocate yoga as a form of daily activity. Home-based yoga training for patients with mild cognitive impairment and Alzheimer's disease not only benefits the patients but also reduces negative emotions in caregivers, such as stress, depression, and anxiety (49, 50). Therefore, yoga can be used as a complementary therapeutic strategy to improve patients with cognitive impairment in PD, and their caregivers can benefit from the accompanying practice.

## Dance

Dance is emerging as an effective intervention for treating a range of PD symptoms, including improving gait, balance, and coordination (51, 52). Dance may also improve cognitive and psychological symptoms, possibly because the execution of dance patterns involves motor planning and memory, and dance provides an opportunity to practice multitasking (51, 53). For example, dancing involves conscious movement and concentration in sync with accompanying music while ensuring postural stability. Additionally, dancers are encouraged to express their feelings and capitalize on emotions, which can increase motivation, provide fun, and potentially improve mood and quality of life (54). Therefore, dance may simultaneously affect motor function, cognitive function, and psychiatric symptoms in people with PD.

A 12-week study by Hashimoto et al. (55) found that dance was effective in improving motor function, cognitive function, and psychiatric symptoms in people with PD. In their study, 46 patients with mild-to-moderate PD were randomly assigned to dance, PD exercise, or no intervention. Dance and PD exercise groups performed weekly 60-min sessions. The patients in the control group continued their normal life. Cognitive function was assessed with the Bedside Frontal Assessment Battery and Mental Rotation Task. Another study by Kalyani et al. (56) explored whether a dance program called Dance for PD® could improve cognition, psychological symptoms, and quality of life in people with PD. This dance session may have a positive effect on memory, as it requires participants to learn motor sequences, store them in memory, and recall and execute spatial poses, relationships, patterns, and paths. The results of the study showed that after the intervention, cognitive skills (executive function and episodic memory) improved significantly in both groups, but no significant differences were shown between groups. Thus, dance classes had significant benefits on PD patients' psychological symptoms and quality of life, but the cognitive benefits were limited. However, the subjects included in this study had a good general cognitive function and were well educated, reducing the scope for improvement. Similar to the study by Michels et al. (57), 10-week dance practice did not improve MoCA scores in people with PD. It may be that the frequency of interventions in this study was limited to weekly. More frequent or longer interventions may allow researchers to detect trends in cognitive improvement. Most dance studies involve twice-weekly sessions of 60–90 min in duration for 6–12 weeks (51). Another study suggested that Argentine tango improves balance and functional mobility, and may have modest benefits for cognition and fatigue in PD. These findings await further confirmation in long-term trials (58).

The primary goal of PD rehabilitation is often to restore motor function, but non-motor symptoms are critical for improving the patient's quality of life. Non-exercise benefits of dance have been previously described for cognition, depression, apathy, and quality of life (59). Dance as a mind-body exercise encourages participants to connect movements to their emotional experiences, enhancing cognitive stimulation (60). The current study lacks direct evidence that dance improves cognitive impairment associated with PD, which may be closely related to the small sample size and insufficient intervention period. Furthermore, there are various forms of dance, and more research is needed to explore the best form for people with PD to participate.

## Limitations

The current study has several limitations. First, mind-body exercises have been proven to improve cognitive impairment in normal elderly, but there is less direct evidence to improve cognitive impairment associated with PD. The sample size of the current study is small and may not accurately represent the effects of mind-body exercises on cognitive abilities in people with PD. Controlled studies with larger sample sizes should be completed in the future to increase the statistical power of the intervention results. Mind-body exercises require meditation and breathing, and these variables may affect outcome measures as these are difficult to control in actual training. Besides, people with PD generally take medicine or training at home to control the disease, and the compliance of patients with independent training in the home environment deserves long-term follow-up and investigation. A systematic review and meta-analysis found that only half of the studies reported adherence information. However, for moderate to long-term interventions (16 weeks−1 year), adherence among eligible individuals was rather low (56.4–66.7%). Dropout rates in each group ranged from 6.3 to 20.8% (61). The most common reasons for withdrawal were personal medical problems, and lack of transportation support (62). There are various modes of mind-body exercises, and there is a lack of unified standard movements, which is not conducive to promotion. The underlying reasons for the improvement of cognitive impairment in PD by mind-body exercises are still unclear, and in-depth mechanism research is lacking. With the development of gut microbiome, metabolomics, PET-CT, and other technologies and equipment, it is helpful to explore the mechanism of mind-body exercises to improve cognitive impairment.

## Conclusion

Cognitive impairment can occur at any stage of PD, and the basic rule is that it is proportional to disease severity (63). Longitudinal studies have shown that more than half of people with early PD show cognitive decline within 5 years, and a quarter of people with PD with mild cognitive impairment develop dementia within 3 years (64, 65). Therefore, the rehabilitation of PD with cognitive impairment is a long-term challenge, and there is an urgent need to explore appropriate and effective therapies to enhance PD patients' cognitive function. Drug therapy can only relieve PD symptoms but does not play a substantial therapeutic role. There is substantial evidence that long-term exercise can delay PD progression (66). A cross-sectional study showed that 12-months of physical activity was inversely associated with cognitive decline (67). Mind-body exercises can improve cognition, executive function, learning, memory, and language fluency (68). Current evidence suggested that physical and mental exercises such as Tai Chi, Qigong, yoga, and dance can be recommended strategies for improving cognitive function in PD patients with cognitive impairment. For mind-body exercise to truly become a useful and acceptable strategy for improving cognitive impairment in people with PD, researchers need to develop clear guidelines for movement patterns to accommodate different severities of cognitive impairment.

## Author contributions

SG conceived the manuscript and revised the drafts. TZ and WL wrote the first draft. All authors contributed to the article and approved the submitted version.

## Funding

This work was supported by the Sports and Health Laboratory Fund Project of Guangxi University of Science and Technology (No. GKDTYSYS2006).

## Conflict of interest

The authors declare that the research was conducted in the absence of any commercial or financial relationships that could be construed as a potential conflict of interest.

## Publisher's note

All claims expressed in this article are solely those of the authors and do not necessarily represent those of their affiliated organizations, or those of the publisher, the editors and the reviewers. Any product that may be evaluated in this article, or claim that may be made by its manufacturer, is not guaranteed or endorsed by the publisher.
